# Effects of Changes in Smoking Behavior of Older Adults’ Oral Health

**DOI:** 10.3390/healthcare10112127

**Published:** 2022-10-26

**Authors:** Kyuhyoung Jeong, Sunghwan Cho, Juhyun Ryu, Heeran J. Cho, Sunghee Kim

**Affiliations:** 1 Department of Social Welfare, Semyung University, Jechon 27136, Korea; 2 School of Social Work, Virginia Commonwealth University, Richmond, VA 23223, USA; 3 Interdisciplinary Graduate Program in Social Welfare Policy, Yonsei University, Seoul 03722, Korea; 4 Department of Health and Welfare, Yuhan University, Bucheon 14780, Korea

**Keywords:** oral health, longitudinal studies, smoking, aged, dental health surveys

## Abstract

While smoking is a crucial health risk, it adversely affects oral health, particularly becoming riskier for older adults who have smoked for a long time. This study identifies the patterns of smoking behavior changes in older adults aged 65 years and older and examines how the smoking behavior changes affect their oral health. Longitudinal data were derived from Korean Longitudinal Study of Aging (KLoSA) between 2006 and 2018, targeting the older adults 65 years and older in South Korea. The independent variable was the amount of smoking, and the dependent variable was oral health. For data analysis, SPSS 25.0 and M-plus 8.0 programs were utilized. As a result, the patterns of changes in smoking behavior of the older adults finally derived were ‘non-smoking,’ ‘decreasing,’ ‘high-level maintenance,’ and ‘decreasing after increasing.’ Furthermore, the relationship between the smoking behavior change pattern of the older adults and oral health was revealed. Specifically, as for the smoking behavior change pattern of the older adults, it was confirmed that the oral health was better in the ‘non-smoking’ pattern compared to the ‘high-level maintenance’ pattern. On the other hand, it was confirmed that the ‘decreasing’ pattern and the ‘decreasing after rising’ pattern did not significantly affect oral health. The findings imply that even if older adults smoked a lot in the past, if they quit smoking at some point, it can positively affect their oral health. Furthermore, it is suggested to allow public officials, medical professionals, and welfare experts to actively intervene for older adults to stop smoking for their oral health.

## 1. Introduction

Smoking is the most critical health risk affecting individuals worldwide [1]; it is reported that smoking causes more than 5 million deaths yearly in the world [2]. Furthermore, smoking is closely associated with cancer, coronary artery disease, Alzheimer’s disease, stroke, and decreased bone mineral density. These risks significantly increase for older adults [3,4], as their immune systems are weakened by smoking and further suppressed accordingly [5]. It has been reported that smoking increases the incidence and mortality of diseases at a rate of about 70% of the population over 60 years of age [6].

Along with smoking, oral health is a common risk factor for geriatric diseases. Poor oral health has a high level of tooth loss and adverse effects on oral cancer, periodontal disease prevalence, and the quality of life of older adults. Extensive tooth loss and performance loss lead to poor eating habits, weight loss, general ill health, and social impairments related to self-esteem and communication about discolored or damaged teeth [7]. It is considered a risk factor with a significant impact on the quality of life accordingly [8,9,10,11]. There is also evidence that the factors affecting oral health are demographic characteristics such as gender and social and economic environments such as household income, residential area, and whether or not they were living alone [12,13,14].

Meanwhile, it has been epidemiologically reported that the lungs have the highest relative risk from smoking, followed by the oral cavity [15]. Smoking causes various diseases by depositing nicotine in periodontal tissue. It is considered as a potential mechanism for increased susceptibility to diseases such as periodontal inflammation, periodontal pockets, and periodontal membrane damage [16]. Smoking also has significant short- and long-term effects on implant treatment outcomes, leading to inflammation and persistent pain [17], caries, halitosis, and oral hygiene [18]. Furthermore, it is confirmed that smoking is a strong predictor of oral diseases such as periodontal disease, oral cancer, tooth loss, and implant failure [16]. Empirically, Gandini and colleagues [17] analyzed twelve studies that estimated the risk of oral cancer in the United States, Italy, China, and South Korea. It reported that the risk estimate of smokers was 3.43 times higher than that of non-smokers.

The scientific evidence linking smoking and oral health in older adults is robust [11], and it is necessary to investigate the definite patterns of smoking behavior changes in the elderly population. While previous studies primarily investigated the relationship between smoking and specific variables such as smoking and dementia [19], cognitive decline [20] and oral health [7], they have focused on measuring and analyzing variables only in the direction of verifying the priori hypothesis.

The relationship between smoking and oral health is often reported contradictory [21], and considerable heterogeneity within the population may be one of the reasons. Furthermore, it is pointed out that it is not possible to estimate different parameters as the change trajectory within the group is presumed to be a single pattern, whether the data are used from one or multiple time points. In other words, presenting in-depth implications might become limited as group heterogeneity cannot be reflected. Considering the conflicting results on the causal relationship between smoking and oral health, the growth-mixed model to establish a group-based trajectory identifies various inconspicuous patterns and takes adequate measures according to intensive review by pattern.

While some studies have tried to identify long-term smoking behavior patterns in South Korea, verifying patterns of smoking behavior change over time would be beneficial. Thus, this study examines the relationship between smoking behavior change patterns and oral health among older adults aged 65 and over in South Korea based on Korean Longitudinal Study of Aging (KLoSA) data from 2006 to 2018.

## 2. Methods

### 2.1. Data

This study analyzed the data of the first to seventh Korean Longitudinal Study of Aging (KLoSA), which were collected by the Korea Employment Information Service (KEIS) from 2006 to 2018. KLoSA handled informed consents, and they obtained them from all participants. The KLoSA is a nationally representative panel survey on the elderly population in South Korea, which aims to produce primary data to establish effective socio-economic policies by measuring and understanding older adults’ social, economic, psychological, demographic, and health status. In this study, the older adults 65 years or older at the time of the first period were targeted, and 1879 people who could estimate their smoking behavior from the first to the seventh period were considered for the final analysis.

### 2.2. Variables

The independent variable was the amount of smoking. In this study, smoking behavior means the number of cigarettes smoked per day (unit: cigarette/day). The dependent variable was oral health, which is identified based on GOHAI (Geriatric Oral Health Assessment Index) developed by Atchison and Dolan [22] and adapted by Shinseon-Jung and Jeong Se-Hwan [23]. The GOHAI consists of twelve items, which reflect those aspects considered to affect the oral health of older adults, such as functional limitation, aesthetic dissatisfaction, chewing discomfort, avoidance of certain food, avoidance of social contacts, and self-medication for dental pain. Each item is based on a 6-point scale (1 = always, 2 = very often, 3 = frequently, 4 = sometimes, 5 = rarely, 6 = never). A higher GOHAI score indicates a higher quality of oral health. Cronbach’s alpha of all items was 0.853.

Control variables were gender, age, household income, residential area, and whether they lived alone. Gender was divided into males and females, and age and household income were continuous variables. Logarithms were taken for a normal distribution of household income. Residential areas were divided into urban and rural areas, and whether they lived alone was divided into living alone and not living alone.

### 2.3. Statistical Analysis

For the data analysis, SPSS 25.0 and M-plus 8.0 programs were used, and the analysis methods and procedures are as follows. First, descriptive statistical analysis was carried out to identify the demographic characteristics of the analysis target and the characteristics of major variables. Second, a potential growth model was performed assuming that groups are classified as a single group to estimate the overall change in smoking behavior. The model’s goodness of fit was tested based on TLI (Tucker–Lewis Index), CFI (Comparative Fit Index), and RMSEA (Root Mean Square Error of Approximation), which are not sensitive to the sample size and considering the simplicity of the model. Third, a Growth Mixed Modeling (GMM) was conducted to classify smoking behavior patterns. In GMM, the optimal number of smoking change patterns was determined through the *p* values of Akaike’s Information Criteria (AIC), Bayesian Information Criteria (BIC), Sample-Size Adjusted BIC (SABIC), Entropy, and Bootstrapped Likelihood Ratio Test (BLRT). Fourth, the χ2-test and one-way ANOVA were conducted to confirm the socio-demographic characteristics of each pattern of change in smoking behavior in the older adults. Fifth, multiple regression analysis was conducted to confirm the effect of smoking behavior change patterns on oral health in older adults.

## 3. Results

### 3.1. Descriptive Statistics

As for the demographic characteristics, the study sample was composed of 736 males (39.2%) and 1143 females (60.8%), and their average income was USD 14,212.9 (SD = 19,243.13). As for the residential area, 1258 people (67.0%) lived in the urban areas and 621 people (33.0%) in the rural areas. In total, 566 people (30.1%) were found to be living alone and 1313 people not living alone (69.9%).

As a result of descriptive statistical analysis of major variables (Table 1), the average smoking amount was 1.80 cigarettes (SD = 5.60) in the first year (2006), 1.59 cigarettes (SD = 5.08) in the second period (2008), 1.33 cigarettes (SD = 4.41) in the third period (2010), 1.32 cigarette (SD = 4.41) in fourth period (2012), 1.06 cigarette (SD = 3.91) in the fifth period (2014), 0.72 cigarette (SD = 3.25) in the sixth period (2016), 0.50 cigarettes (SD = 2.74) in the seventh period (2018). It depicted a tendency of gradual decrease as time passed. For oral health, the average score was 3.84 (SD = 0.74) out of 6. As a result of examining the normality of the main variables, skewness was less than 3 in absolute value, and kurtosis was less than 10 in absolute value, indicating that the distribution of variables did not deviate from normality [24].

### 3.2. Smoking Behavior Change and Typology

Before proceeding with the GMM, a latent growth model was performed to understand the older adults’ overall change in smoking behavior (Table 2). For this purpose, the no-change model, the linear change model, and the quadratic function change model were analyzed and compared the model’s goodness of fit, respectively. As a result of the analysis, the no-change and linear change models were discovered to be unsuitable. On the other hand, the quadratic function change model was adopted satisfying goodness of fit with values of χ^2^ = 67.020, CFI = 0.824, TLI = 0.934, and RMSEA = 0.091.

As a result of estimating the GMM based on the quadratic function change model, the goodness of fit for the four classes’ pattern classification was revealed to be lower for AIC, BIC, SABIC, and Entropy, closer to 1 compared to the classification of one class, two classes, and three classes’ patterns classification (Table 3). In addition, two classes, three classes, and four classes’ patterns of BLRT were significant. Therefore, the four classes’ pattern of smoking behavior change of the older adults were judged to be the most suitable and adopted as the final model when the goodness of the model fit criteria was comprehensively considered.

**Figure 1:**
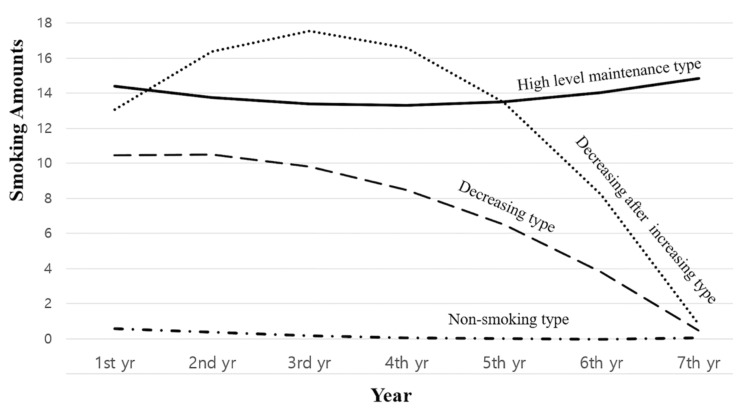
Estimation of smoking behavior change patterns in the older adults.

Changes in smoking behavior of older adults in the adopted model were classified into four patterns, and the name of each pattern was set, reflecting the characteristics of the change in smoking behavior (Figure 1). The first pattern was named the ‘non-smoker’ class, as the number of cigarettes smoked per day from the first year to the seventh year was almost zero, and 1693 cases (90.1%) were classified as this pattern. In the second pattern, the number of cigarettes smoked per day was about ten cigarettes in the first year and almost 0 in the seventh year, so it was named a ‘reduced’ class, and 82 cases (4.4%) were classified as this pattern. The third pattern was designated as the ‘high-level maintenance pattern’ class as it was found that the number of cigarettes smoked per day was maintained at about 14 cigarettes from the first to the seventh year, and 53 cases (2.8%) were classified as this pattern. In the fourth pattern, the number of cigarettes smoked per day increased from about thirteen in the first year to sixteen in the third year and was nearly zero in the seventh year. Thus, it was named the ‘rising and decreasing’ class, and one-fifth of cases (2.7%) were classified into this pattern.

### 3.3. Differences in Demographic Characteristics by Patterns of Changes in Smoking Behavior

As a result of examining the differences in demographic characteristics by a pattern of change in smoking behavior in the older adults, gender (X^2^ = 212.705, *p* < 0.001), age (F = 4.675, *p* < 0.01), and whether they were living alone or not (X^2^ = 20.957, *p* < 0.001) showed a significant difference (Table 4). In terms of gender, it was confirmed that the ‘non-smoking’ pattern had a higher proportion of females, and other patterns had a higher proportion of males. In terms of age, the ‘non-smoking’ pattern and the ‘decreasing’ pattern were significantly higher than the ‘high-level maintenance’ pattern. Regarding whether they lived alone or not, the rate of the older adults not living alone was higher than those living alone, but the rate of living alone was about twice as high for the ‘non-smoking’ pattern than for other patterns.

### 3.4. The Relationship between Older Adults’ Smoking Behavior Change Patterns and Oral Health

Multiple regression analysis was performed to verify the relationship between the smoking behavior change pattern of the older adults and oral health (Table 5). The explanatory power of oral health was 16.7% (R^2^ = 0.167), and the research model was confirmed to be suitable (F = 11.659, *p* < 0.001). Looking at the results of the analysis, in the control variables, gender (β = −0.116, *p* < 0.001) and age (β = −0.170, *p* < 0.001) were found to effect on oral health. In other words, it was analyzed that the oral health was better for men and younger age. On the other hand, household income, residential areas, and whether or not they were living alone had no significant effect on oral health.

As for the smoking behavior change pattern of the older adults, it was confirmed that the oral health-related to quality of life was better in the ‘non-smoking’ pattern compared to the ‘high-level maintenance’ pattern (β = −0.060, *p* < 0.01). On the other hand, it was confirmed that the ‘decreasing’ pattern and the ‘decreasing after rising’ pattern did not significantly affect oral health.

## 4. Discussion

This study recognizes the patterns of smoking behavior changes in older adults 65 years and older and reveals how the derived smoking behavior changes affect the oral health. For this purpose, the secondary data are derived from the KLoSA between 2006 and 2018, targeting the older adults 65 years and older in South Korea.

The main results and suggestions of this study are as follows. The patterns of changes in smoking behavior of the older adults finally derived are ‘non-smoking’, ‘decreasing’, ‘high-level maintenance’, and ‘decreasing after increasing’. The academic significance of this study is that it longitudinally confirmed the changes in smoking behavior of older adults over 65 and derived specific actual patterns.

As a result of examining the effects of different patterns of smoking behaviors on oral health, the high-level maintenance pattern is found to be very vulnerable compared to that of the non-smoking pattern. This correspond well with previous studies that smokers are more likely to be exposed to oral diseases than non-smokers [4,25], and the studies that revealed smoking as one of the crucial factors influencing changes in the oral microbial ecosystem at the biological level [26]. It should be noted that there is no difference in the oral health between the decreasing pattern and the decreasing after increasing pattern compared to the non-smoking pattern. In other words, even if individuals smoke a lot, if they quit smoking at some point, it can positively affect their oral health. In the case of smoking in old age, there is a false myth that damage is done because it is already done, and that quitting smoking does not make them healthy [27]. However, the results of this study show that this perception is merely a prejudice.

Furthermore, some studies support that smoking cessation in old age help to prolong life [28], lowers the danger of developing lung cancer [29] or dementia [30], and functions as a protective factor against suicidal thoughts [31]. They maintain that smoking cessation benefits older adults’ physical and mental health beneficially. It is never too late to quit smoking, considering it a risk factor for the social cost of various diseases and the oral health of older adults. If the smoking change behavior is a high-level maintenance pattern, immediate intervention is required as the oral health is considered to be very low. However, practical and policy efforts on tobacco control mostly focus on the young population, such as children and adolescents, and those in their 20s, 30s, and 40s [32]. In South Korea, public health centers operate smoking cessation clinics nationwide, and various smoking cessation support services such as smoking cessation counseling lines and support for smoking cessation treatment in hospitals [33]. However, there are limitations in that it is premised on a decision to quit smoking and a smoking cessation plan. There is a necessity for active smoking cessation interventions for older adults. To this end, policy and practical efforts should be supported, rather than merely waiting for those who want to quit smoking. Accordingly, older adults can recognize and participate in the importance of smoking cessation on their own. Specifically, it is crucial to encourage older adults to quit smoking. This can be carried out by allowing public officials, medical professionals, and welfare experts, who frequently interact with older adults, to recommend the importance of quitting smoking in old age and suggest methods they can apply without considerable barriers.

While this study has academic significance as it derives the patterns of changes in the smoking behavior of older adults and identifies the relationship with oral health, it has the following limitations. First, older adults who died in the middle of the longitudinal study were excluded from the subject of the study. It means that older adults who did not survive the 12 years of the study period were all excluded, which might be quite a lot as the subject of this study is the older adults. Accordingly, it is recommended to set a shorter survey period for future studies to examine the relationship between smoking behavior and the section health of older adults. Second, this study measured oral health-related data only in the seventh year. It is expected that the relationship between smoking behavior and oral health can be revealed more evidently if oral health is measured for a more extended period.

## 5. Conclusions

While smoking in the elderly significantly impacts oral health, long-term disease, and mortality, derived smoking behavior patterns allow us to understand older adults’ smoking behavior and oral health based on divided patterns. In conclusion, oral health is better in the smoking behavior of the ‘non-smoking’ pattern compared to the ‘high-level maintenance’ pattern. The ‘decreasing’ pattern and the ‘decreasing after rising’ pattern do not significantly affect oral health, implying that even if older adults smoked a lot in the past, if they quit smoking at some point, it can positively affect their oral health. Understanding the smoking behavior patterns’ characteristics may lead to more efficient and effective public intervention for older adults’ oral health.

## Figures and Tables

**Table 1:** Descriptive statistics (*n* = 1879).

Variable	Min	Max	M	SD
Amount of Smoking in the first year	0	60	1.80	5.60
Amount of Smoking in the second year	0	40	1.59	5.08
Amount of Smoking in the third year	0	40	1.33	4.41
Amount of Smoking in the fourth year	0	40	1.32	4.41
Amount of Smoking in the fifth year	0	30	1.06	3.91
Amount of Smoking in the sixth year	0	30	0.72	3.25
Amount of Smoking in the seventh year	0	40	0.50	2.74
Oral Health	1.33	6.00	3.84	0.74

Note: Min = minimum, Max = maximum, M = mean, SD = standard deviation.

**Table 2:** Model fit of Latent Growth Modeling for Older Adults’ Smoking Behavior Changes.

Model	χ^2^	CFI	TLI	RMSEA
No growth model	3244.129 ***	0.682	0.743	0.257
Linear model	165.159 ***	0.824	0.839	0.142
Quadratic model	67.020 ***	0.940	0.934	0.091

Note: χ^2^ = chi-square, CFI = Comparative Fit Index, TLI = Tucker–Lewis Index, RMSEA = Root Mean Square Error of Approximation, *** *p* < 0.001.

**Table 3:** Goodness of Fit for GMM (*n* = 1,879).

Class	Model Fit					Groups
	AIC	BIC	SSABIC	Entropy	BLRT *p*-Value	N (%)
1	68,897.689	69,013.997	68,947.280	-	-	-
2	66,207.714	66,351.715	66,269.114	0.922	<0.001	1823 (97.0), 56 (3.0)
3	64,304.031	64,475.724	64,377.238	0.945	<0.001	1722 (91.6), 103 (5.5), 54 (2.9)
4	63,698.785	63,898.171	63,783.799	0.997	<0.001	1693 (90.1), 82 (4.4), 53 (2.8), 51 (2.7)

Note: AIC = Akaike’s Information Criteria, BIC = Bayesian Information Criteria, SABIC = Sample-Size Adjusted BIC, BLRT = Bootstrapped Likelihood Ratio Test.

**Table 4:** Differences of Patterns of Older Adults’ Smoking Behavior Changes (*n* = 1879).

Variable		Non-Smoking Pattern		Decreasing Pattern		High-Level Maintenance Pattern		Decreasing after Increasing Pattern		X^2^/F
		*n*	%	*n*	%	*n*	%	*n*	%	
Gender	Male	571	33.7	72	87.8	47	88.7	46	90.2	212.705 ***
	Female	1122	66.3	10	12.2	6	11.3	5	9.8	
Age	Mean (SD)	82.6 ^a^ (4.64)		82.7 ^b^ (4.05)		80.5 ^c^ (3.52)		81.5 (4.33)		4.675 **
Household Income	Mean (SD)	14,419.6 (19962.24)		11,363.3 (9237.72)		14,249.7 (12825.78)		11,893.6 (9689.52)		0.911
Residential areas	Urban	1142	67.5	45	54.9	38	71.7	33	64.7	6.251
	Rural	551	32.5	37	45.1	15	28.3	18	35.3	
Whether living alone or not	Living Alone	537	31.7	13	15.9	7	13.2	9	17.6	20.957 ***
	Not Living alone	1156	68.3	69	84.1	46	86.8	42	82.4	

Note: t = t-statistic, F = F-statistic, SD = standard deviation, ** *p* < 0.01, *** *p* < 0.001, Scheffe’s test: ^a^, ^b > c^.

**Table 5:** Multiple Regression Analysis for Oral Health (*n* = 1879).

Variables	B	S.E.	β
(Constant)	6.169	0.327	
Gender (0 = Male)	−0.044 ***	0.010	−0.116
Age	−0.027 ***	0.004	−0.170
Household Income (ln)	0.023	0.015	0.037
Residential Area (0 = Urban Area)	−0.043	0.036	−0.027
Whether or Not Living Alone (0 = Not Living Alone)	−0.010	0.040	−0.006
Smoking Behavior: Decreasing Pattern (0 = Non-smoking Pattern)	−0.170	0.103	−0.038
Smoking Behavior: high-level maintenance pattern (0 = Non-smoking Pattern)	−0.219 **	0.084	−0.060
Smoking Behavior: Decreasing after Increasing Pattern (0 = Non-smoking Pattern)	−0.096	0.105	−0.021
R^2^	0.167		
F(sig.)	11.659 ***		

Note: B = regression coefficient, S.E. = standard error, β = standardized coefficients, ** *p* < 0.01, *** *p* < 0.001.

## Data Availability

This study used a secondary data from KLoSA (https://survey.keis.or.kr/eng/klosa/klosa01.jsp, accessed on 6 October 2022). These data are open to public for research purposes.
